# On the identity of the tamarin AMNH 98303 (“*Saguinus fuscicollis tripartitus*”; Primates: Haplorrhini: Simiiformes: Platyrrhini: Callitrichidae)

**DOI:** 10.5194/pb-9-1-2022

**Published:** 2022-01-24

**Authors:** Eckhard W. Heymann

**Affiliations:** Verhaltensökologie & Soziobiologie, Deutsches Primatenzentrum, Leibniz-Institut für Primatenforschung, Kellnerweg 4, 37077 Göttingen, Germany

## Abstract

The American Museum of Natural History houses the skin of a tamarin (AMNH 98303) labelled as *Saguinus fuscicollis tripartitus*. However, the specimen does not match the phenotype of this taxon, now named *Leontocebus tripartitus*, nor that of any other known species or subspecies of *Leontocebus*. In this note, we review past taxonomic revisions of the genus *Saguinus* – revisions that were largely driven by the contentious species or subspecies status of the golden-mantled saddleback tamarin *S. fuscicollis tripartitus* – and compare the phenotype of AMNH 98303 with those of other tamarins in the same genus to discuss the possible status of this specimen.

## 1 Introduction: tamarin taxonomy and the status of the taxon *tripartitus*

Tamarins are a diverse group of New World primates with a synonymic history of more than nine genera. All were placed in the genus *Saguinus* Hoffmannsegg by Hershkovitz (1977), but Rylands et al. (2016) separated out Hershkovitz's *Saguinus* *nigricollis* or white-mouthed tamarin group as belonging to the genus *Leontocebus* Lesson. Two species comprised Hershkovitz's *S. nigricollis* group (the black-mantled tamarin *Saguinus* *nigricollis* and the saddleback tamarin *Saguinus* *fuscicollis*), and Hershkovitz (1966, 1977) placed the golden-mantled saddleback tamarin *S. fuscicollis tripartitus* Milne-Edwards, 1878, as a subspecies of *S. fuscicollis*. Considered by Hershkovitz (1977) as a subspecies, Thorington (1988) elevated *S. fuscicollis tripartitus* to species rank, *Saguinus tripartitus*, based on an alleged sympatry with *Saguinus fuscicollis lagonotus*. Evidence for this sympatry is, however, absent or at best very weak (reviewed in Rylands et al., 2011). In 2000, I indicated that the lack of evidence for sympatry between the two tamarins meant that tripartitus should be downgraded to a subspecies or that the 13 of the 14 subspecies of *S. fuscicollis* of Hershkovitz (1977) considered valid at the time be considered speciesIn 1996, Peres et al. showed that *Saguinus fuscicollis acrensis* was a hybrid.. Molecular studies subsequently revealed that *S. fuscicollis tripartitus* clusters in a clade comprising several subspecies of *S. fuscicollis* (Matauschek et al., 2011; Buckner et al., 2015), which would have made this clade paraphyletic if the species status of *S. fuscicollis tripartitus* or the subspecies status of the other taxa were retained. Finally, this led to a taxonomic revision that resulted in two major changes (Rylands et al., 2016): (1) separation of the small tamarins of Hershkovitz's (1977) *S. nigricollis* group as a genus of its own, *Leontocebus*; (2) elevation of most subspecies of the former species *S. fuscicollis* to species rank.

**Figure 1:**
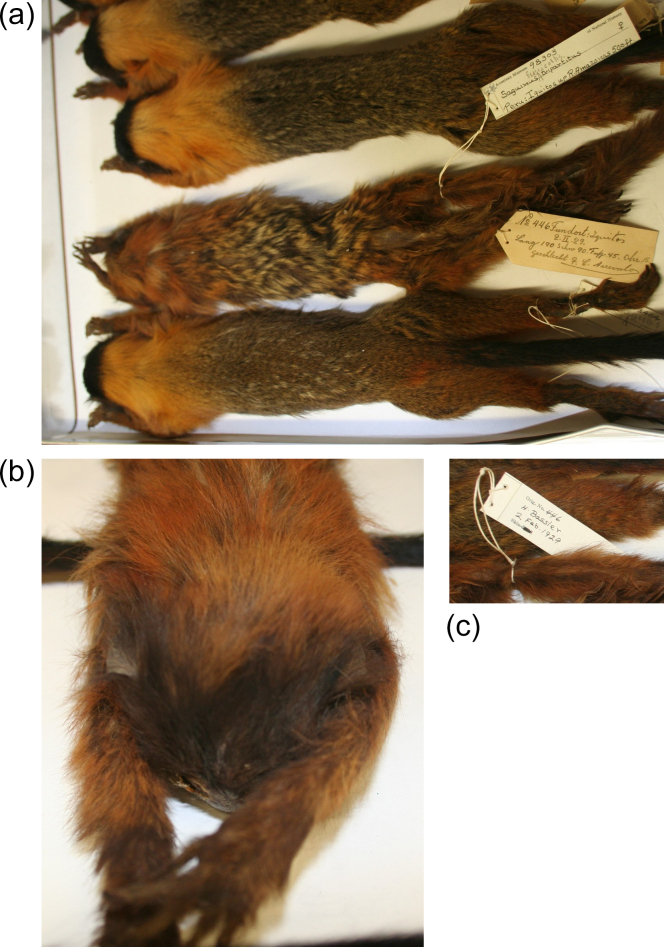
**(a)** Specimens of *Saguinus fuscicollis tripartitus* (above and below) and the doubtful specimen AMNH 98303 (centre). **(b)** Frontal view of AMNH 98303. **(c)** Backside of the tag. Photos: Christian Matauschek.

## 2 The specimen AMNH 98303 and why it cannot be *S. fuscicollis tripartitus*

The American Museum of Natural History houses a number of skins of tamarins, amongst them several labelled as *Saguinus fuscicollis tripartitus*, one of them being AMNH 98303. However, this specimen contrasts very strongly with the other *S. fuscicollis tripartitus* specimens (Fig. 1a, b): it lacks the black forehead and face; the neck is not uniformly orange-golden but rather reddish-brownish; and the back (“saddle”) shows the typical agouti pattern of most members of the *S. nigricollis* group of Hershkovitz (1977). According to the original label tagged to the specimen, it is a female found (German *Fundort*: place where found) in Iquitos by C. Arrevalos on 29 February 1929 (Fig. 1a). A second label, carrying the name H[arvey] BasslerHarvey Bassler worked as chief geologist for Standard Oil from 1921 to 1931 in Peru, with Iquitos being his headquarters (Myers, 2000). During his expeditions in Peruvian Amazonia, he mainly collected amphibians and reptiles, but also some mammals. These collections went to the American Museum of Natural History for which Bassler worked as a research associate at the Department of Herpetology from 1932 onward (Myers, 2000). No information could be found on C. Arrevalo (perhaps a misspelling of Arevalo)., also quotes Iquitos (Fig. 1a, c). The range of the golden-mantled tamarin is restricted to the interfluvium between the rivers Napo and Curaray, while Iquitos is located on the Amazon below its confluence with the Napo; Iquitos can, therefore, only be the place where the tamarin was obtained, probably purchased, by the collector, and not the place of origin.

The phenotype of AMNH 98303 does not match any other species or subspecies of *Leontocebus* in the region around Iquitos (*Leontocebus nigricollis graellsi*, *Leontocebus nigricollis nigricollis*, *Leontocebus lagonotus*, *Leontocebus nigrifrons*, *Leontocebus illigeri*, *Leontocebus fuscicollis fuscicollis*) or any other Peruvian member of the genus (*Leontocebus leucogenys*, *Leontocebus weddelli melanoleucus*, *Leontocebus weddelli weddelli*; see Aquino et al., 2015, and Fig. 1 in Rylands et al., 2016). Superficially, it shows similarity to *Leontocebus cruzlimai*, mainly because of the overall reddish colouration as shown in Rylands et al. (2016: Fig. 1) and da Cruz Lima (1945: Plate 38, Fig. 3a). However, the drawings may not be representative of its phenotype; a photo of a living animal in Sampaio et al. (2015), Fig. 4, does not show the bright reddish colouration. Also, AMNH 98303 lacks the white frontal blaze above the eyes, although this is probably a variable trait. It occurs in some individuals of *L. lagonotus*, a species that otherwise lacks it, being present in some AMNH specimens (Voss and Fleck, 2011) and also observed in the wild (Christian Matauschek, personal communication, 2008). Finally, *L. cruzlimai* is distributed in Brazil between the rivers Purus and Pauini, a considerable distance from Iquitos, making it unlikely (but not impossible) that the animal was taken to Iquitos from there.

With a head–body length (assuming that the German word *Lang* on the label refers to *Länge*, meaning length) of 190 mm, AMNH 98303 is not a fully grown adult. Adults of other *Leontocebus* taxa range from 223 to 232 mm (Rylands et al., 2011; see also measurements in Hershkovitz, 1977). Since there is no evidence in other tamarin species for colouration changes during ontogeny, except for the area around the mouth during the transition from infant to juvenile (Eckhard W. Heymann, personal observations), and since the measurement indicates that the specimen was approaching adulthood, the possibility can be excluded that it had not yet reached “adult colouration”. It therefore differs from *S. fuscicollis tripartitus* and all other recognized white-mouthed *S. nigricollis* group tamarins.

## 3 AMNH 98303: hybrid or unknown species?

Could AMNH 98303 be a hybrid between different *Leontocebus* species? Tamarins are known to hybridize in captivity and in the wild (Hershkovitz, 1977; Peres et al., 1996). At this point it is interesting to note that Encarnación et al. (1990) reported *S. fuscicollis tripartitus* from the south bank of the Río Yuvineto, a right-bank tributary of the Río Putumayo, where it would be sympatric with *L. nigricollis graellsi*. The second half of his description coincides neither with *S. fuscicollis tripartitus* nor with AMNH 98303: “Esta subespecie es fácilmente diferenciable del *S. fuscicollis nigrifrons* por la presencia de la pequeña bifurcación blanca frontal y color agutí intenso de la mitad posterior del cuerpo” (p. 75; “This subspecies can be easily distinguished from *S. fuscicollis nigrifrons* by the presence of a small white frontal bifurcation and the intensive agouti colouration of the posterior half of the body”). But it does also not coincide with the phenotypes of *L. nigricollis graellsi* and *L. nigricollis nigricollis*, whose ranges are parapatric around the area of the Putumayo–Napo interfluvium mentioned by Encarnación et al. (1990) for *S. fuscicollis tripartitus*.

The possibility should not be excluded that AMNH 98303 is an undescribed taxon. Despite a long history of primatological field explorations and collections, 124 new primate species have been described since 1990, 32 of them from the neotropics (Mittermeier and Rylands, 2021), having either been newly discovered in the wild or re-described through examination and genetic analyses of existing museum specimens.

In conclusion, AMNH 98303 is not *Leontocebus tripartitus*. Its real taxonomic status will remain unresolved until pertinent genetic analyses are undertaken. For comparison, mtDNA sequences are available for a large number of *Leontocebus* taxa (Matauschek et al., 2011; Buckner et al., 2015; Sampaio et al., 2015). Stable isotope analyses could also help to explore its geographic origin (Koehler et al., 2019; Dominy et al., 2020). Also, other museums could revise their tamarin collections to see whether they include specimens that phenotypically match AMNH 98303.

This note underlines the value of museum collections for exploring the diversity of life and shows that we are still far from knowing this diversity even in an otherwise rather well-known taxon such as primates.

## Data Availability

No data sets were used in this article.
